# Supplementary material for: Understanding Confounding Effects in Linguistic Coordination: An Information-Theoretic Approach
Source: PLoS One. 2015 Jun 26;10(6):e0130167. doi: 10.1371/journal.pone.0130167 (PMC4483141; doi:10.1371/journal.pone.0130167)
Supplement: S1 File — (PDF) [file pone.0130167.s001.pdf]

S1 Basic Concepts in Information Theory

Consider a random variable X with probability distribution $p(x) \equiv p(X = x)$. In the discrete case, the *Shannon* entropy is defined as:

$$H(X) = -\sum_{x} p(x) \log p(x) \tag{14}$$

Note that If X is a continuous variable, the sum in Eq. 14 is replaced by an integral. We often talk about the entropy $H(X)$ s quantifying our uncertainty about the random variable, so that higher entropy means more uncertain (and less predictable) X. In particular, entropy is zero, $H(X) = 0$, if and only if X is perfectly predictable.

Let us now consider two random variables, X and Y, and let $p(x, y)$ denote their joint distribution. The (joint) entropy for X and Y is defined similarly as

$$H(X, Y) = -\sum_{x}\sum_{y} p(x, y) \log p(x, y) \tag{15}$$

We can also define conditional entropy of Y given X (or vice versa), as follows:

$$H(Y|X) = -\sum_{x} p(x) \sum_{y} p(y|x) \log p(y|x) \tag{16}$$

If X and Y are independent, then their joint entropy is the sum of individual entropies, $H(X, Y) = H(X) + H(Y)$. Indeed, note that for independent X and Y, their joint distribution factorizes as $p(x, y) = p(x)p(y)$. Plugging this factorized distribution into Eq. 15 then yields the additivity of the joint entropy.

In case X and Y are not independent, the degree of their dependence can be measured by *mutual information* (MI), defined as follows:

$$I(X:Y) = H(X) + H(Y) - H(X, Y) \tag{17}$$

$I(X:Y)$ measures the correlation between X and Y, and equals to zero if and only if X and Y are independent. In essence, $I(X:Y)$ measures the amount of uncertainty reduction in X, if we know Y, and vice visa. This intuition becomes more clear if we rewrite the mutual information the in the following form:

$$I(X:Y) = H(Y) - H(Y|X) \tag{18}$$

$$= H(X) - H(X|Y) \tag{19}$$

The relationships between the entropies, conditional entropies, and mutual information are captured in the Venn diagram shown in Fig. A.

For a more detailed account of information-theoretic concepts, we refer the reader to a classical textbook by Cover and Thomas [9].

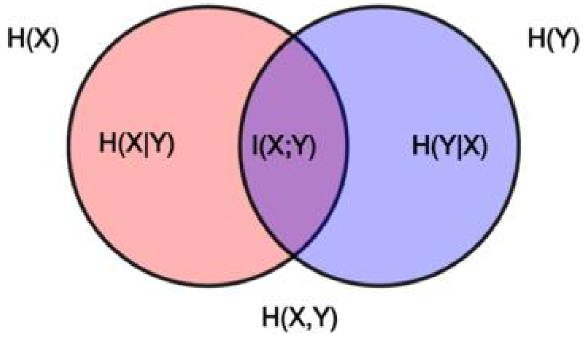

Figure A. The Venn diagram depicting the relationship between individual ($H(X)$, $H(Y)$), joint ($H(X,Y)$), and conditional ($H(X|Y)$, $H(Y|X)$) entropies. The intersection of the circles is the mutual information $I(X;Y)$.
