# Supplementary material for: Understanding Confounding Effects in Linguistic Coordination: An Information-Theoretic Approach
Source: PLoS One. 2015 Jun 26;10(6):e0130167. doi: 10.1371/journal.pone.0130167 (PMC4483141; doi:10.1371/journal.pone.0130167)
Supplement: S2 File — (PDF) [file pone.0130167.s002.pdf]

S2 Stylistic Coordination and Power Relationship

It has been hypothesized that directionality of the stylistic coordination in dialogues can be predictive of power relationship between the conversations, as discussed in [5]. We do indeed observe directional differences in stylistic coordination when comparing Figs. 1(a) and 1(b) with Figs. 2(a) and 2(b). However, as we elaborated above, the observed directionality can result from the confounding effect of length coordination.

Here we analyze this issue in more details by setting up the following prediction task (see [5]). We consider all the pairs of users (X, Y) who have different social status (e.g., admin vs. non-admin) and have engaged in dialogues. We then calculate stylistic coordination scores from X to Y and Y to X, and examine whether those scores can be used to classify the social status of each speaker. For classification, we assume we know the status relationship for a fraction of pairs in our dataset, and then use a supervised learning method called Support Vector Machine (SVM) to predict the status of the unknown users. We perform this prediction tasks using the following three different set of features:

- *Coordination Features*: For each pair, and for each of the eight stylistic markers, we produce two-dimensional feature vector, where the two components correspond to the mutual information score in either direction.

- *Aggregated Coordination Features*: For each pair, we aggregate the *Coordination Features* for all eight stylistic markers in both directions, which results in a sixteen-dimensional feature vector.

- *Length Coordination Features*: For each pair, we calculate the Pearson correlation coefficients between length of utterances in either direction, and use those coefficients as an input to SVM.

For the Wikipedia data, we consider (admin, non-admin) pairs and for the Supreme Court data, we consider (justice, lawyer) pairs.

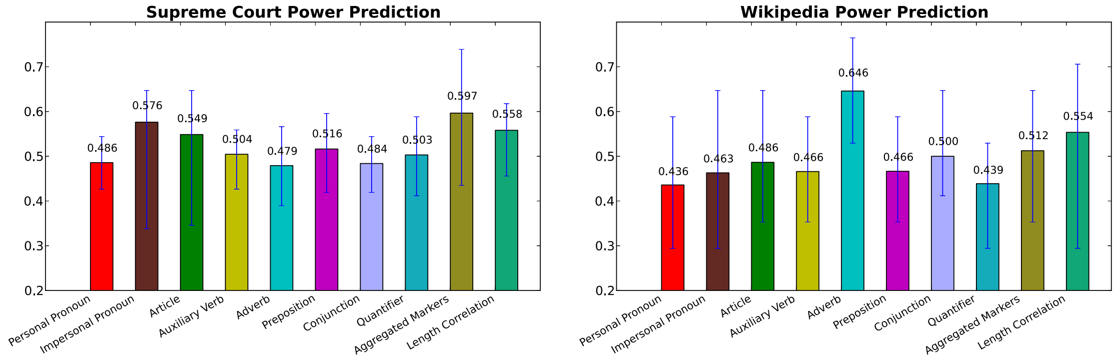

(a) (b)

Figure A. SVM Prediction Accuracy for both stylistic coordination features and length coordination features

In our experiment, we only select pairs which have at least 20 exchanges between them, so that we can calculate mutual information with reasonable accuracy. This results in 135 pairs in Supreme Court dataset and 34 pairs in Wikipedia dataset. Also, we labeled half fraction of the pairs, shuffled the training data and repeated the procedure $N = 100$ times to calculate the average prediction accuracy.

Fig. A depicts the prediction accuracy for each of the above scenario, together with error bars which give the 95% confidence intervals. Since the two datasets are small, the error bars are relatively large in these situations. The results can be summarized as follows. For the Supreme Court dataset, the best

prediction accuracy is achieved when using *Aggregated Coordination Features*, whereas for the Wikipedia dataset the best accuracy corresponds to using coordination on the feature *Adverb*. More importantly, we find that using *Length Coordination Features* alone can predict user status with better-than-random (50%) accuracy. In fact, investigating from the error bars, we cannot rule out that the hypothesized correlation between social status and (the direction of) stylistic coordination is due to the confounding effect of length coordination.
